# A Unilateral Intratonsillar Abscess in an Adult Treated by Antibiotic Therapy and Needle Aspiration

**DOI:** 10.7759/cureus.67060

**Published:** 2024-08-17

**Authors:** Ku Qayrunnisa Ku Ismail, Atikah Rozhan, Wan Ishlah Leman

**Affiliations:** 1 Otolaryngology and Head and Neck Surgery, International Islamic University Malaysia, Kuantan, MYS

**Keywords:** tonsillectomy, computed tomography scan, incision and drainage, antibiotics therapy, needle aspiration, tonsillitis, peritonsillar abscess, intratonsillar abscess

## Abstract

Intratonsillar abscess (ITA) is a rare clinical phenomenon in pediatric and adult populations. Even though it is rather uncommon, ITA should be included in the differential diagnosis of tonsillitis, peritonsillitis, and peritonsillar abscess. A computed tomography (CT) scan serves as a diagnostic tool for confirming the presence of an ITA. Needle aspiration of the tonsil not only further confirms the diagnosis but as part of the treatment of ITA in this case. Here, we report a case of a 34-year-old gentleman who presented with acute painful right neck swelling associated with odynophagia, voice changes, and reduced oral intake with examination revealed muffled voice, trismus, right cervical lymphadenopathy, bulging of the right peritonsillar region and deviation of the uvula to the left. Intravenous antibiotic therapy started and proceeded with aspiration then incision and drainage of the right peritonsillar region done as the initial provisional diagnosis was a right peritonsillar abscess. However, no pus drained. Thus, contrast-enhanced computed tomography (CECT) of the neck was done to assess the abscess collection and confirm the diagnosis. Therefore, the diagnosis was revised to the right ITA as the CECT neck showed a hypodense collection in the right palatine tonsil. In our case, the patient was treated by aspiration of the right tonsil to drain the pus with the continuation of antibiotics. Subsequently, the symptoms improved and resolved. In this case, the ITA can be treated with antibiotics and needle aspiration without surgical intervention.

## Introduction

Intratonsillar abscesses (ITAs) constitute a rare complication arising from a common infection, namely acute bacterial tonsillitis. The palatine tonsils are situated in the anatomical space bounded by the palatoglossal muscle anteriorly, the palatopharyngeal muscle posteriorly, and the superior pharyngeal constrictor muscle laterally [1-3]. The palatine tonsils are surrounded by a non-keratinized squamous epithelium medially and a fibrous sheath of connective tissue laterally. The tonsillopharyngeus contracts because of the activation of the superior pharyngeal constrictors [1]. This contraction facilitates the expulsion of crypt contents. The medial surface of the tonsil comprises multiple tonsillar crypts varying from 8 to 20 in number [1,2]. The clinical presentation may mimic tonsillitis, peritonsillitis, or peritonsillar abscess, necessitating imaging for definitive diagnosis confirmation if uncertain [4]. Treatment modalities for ITAs encompass a spectrum ranging from intravenous antibiotic administration and needle aspiration to incision and drainage and in some instances, tonsillectomy [3]. In our case, needle aspiration with intravenous antibiotic coverage as the primary treatment modality is effective without surgical intervention. We report the case of a 34-year-old man with a right unilateral ITA.

## Case presentation

A 34-year-old male, an active smoker without known medical comorbidities, presented with a four-day history of sore throat accompanied by fever. Additionally, he reported a two-day onset of painful right neck swelling, odynophagia, voice changes, and reduced oral intake. The patient had a single episode of tonsillitis one year prior, necessitating antibiotic treatment without hospitalization. Upon examination, the patient exhibited no respiratory distress or audible stridor. Clinical findings included a "hot potato" voice and trismus with a tender, warm, mobile, and firm right cervical lymph node at level II measuring 1 x 2 cm. Oropharyngeal examination revealed bulging of the right peritonsillar region, with the uvula slightly deviated to the left and bilateral tonsils graded III, erythematous without exudate. Flexible nasopharyngeal scope examination yielded normal findings.

**Figure 1:**
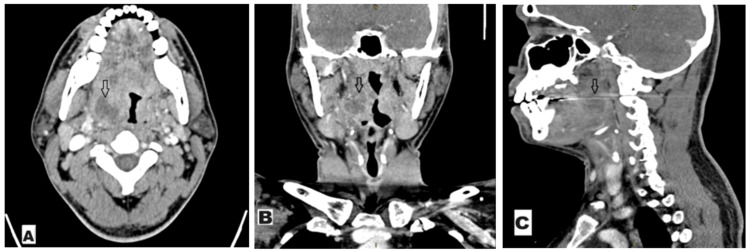
CECT of the neck demonstrating hypodense collection within the right palatine tonsil, measuring approximately 2.0 x 2.2 x 2.7 cm. A) A CECT of the neck axial cut with an arrow showing the right palatine tonsil collection; B) a CECT of the neck coronal cut with an arrow showing the right palatine tonsil collection; C) a CECT of the sagittal cut with an arrow showing the right palatine tonsil collection CECT: contrast-enhanced computed tomography

An attempted aspiration as well as incision and drainage of the right peritonsillar region failed to yield pus. Therefore, proceed with contrast-enhanced computed tomography (CECT) of the neck was performed and revealed an irregular, peripherally enhancing hypodense collection within the right palatine tonsil measuring approximately 2.0 x 2.2 x 2.7 cm (Figures 1A-1C). Blood parameters indicated a white blood cell count of 16.8 x 10⁹, with a neutrophil predominance. This corroborated the diagnosis of a unilateral right ITA.

The patient was started on intravenous Augmentin 1.2 g TDS and received appropriate analgesia. Due to the collection of ITA from the CECT neck, aspiration of the right tonsil was done and yielded 4 cc of pus. Results indicated *Streptococcus pyogenes* growth in the pus culture, while mycobacterial C+S and AFB were negative. The patient completed a four-day course of intravenous Augmentin 1.2 g TDS and was successfully discharged to complete a total of 14 days of treatment. After being discharged, the patient had no active complaints and was in good general condition after one week and one month. He resumed normal oral tolerance, and examinations revealed bilateral tonsils graded III with no peritonsillar bulging. Flexible nasopharyngeal scope findings were unremarkable. He was subsequently discharged from further follow-up.

## Discussion

ITA is defined as a collection of neutrophils and necrotic debris in the parenchyma of the tonsil and happens as a sequela of acute tonsillitis [5]. Meanwhile, the exact etiology of ITA is uncertain, but Michaels and Hellquist et al. in 2001 found that in instances where a suppurative focus develops acute tonsillitis and external drainage is impeded due to crypt blockage resulting in accumulation of pus may intrude inward and potentially culminate in the formation of an ITA [6]. The inability of the tonsillopharyngeus muscle to effectively clear debris and food coupled with subsequent localized inflammation is a contributing factor in the studies [7]. Besides that, ITA can occur due to bacterial seeding by the lymphatic system or bloodstream [8]. The lymphatic transport from the palatine tonsils together with the absence of lymphatic valves preceding the capsule may hinder the aggregation of bacteria within the tonsillar parenchyma [5]. This phenomenon may contribute to the comparatively low incidence of ITA in contrast to the more prevalent occurrence of peritonsillar abscess. Factors compromising lymphatic flow such as dehydration, inflammatory swelling of follicles, and a history of peritonsillar abscess are postulated as predisposing factors for ITA [3].

In terms of tonsil bacteriology, the most common pathogen that has been cultured in both adults and children is *Staphylococcus aureus*. Enterobacter and *Escherichia coli* are commonly isolated in adults while *S. pyogenes* is more common in children [2,3]. In our case, the culture was *S. pyogenes*, and the patient responded well to the antibiotics. Peritonsillar abscesses can usually be diagnosed through oral examination, so computed tomography (CT) is often not needed for diagnosis. However, if the symptoms do not improve despite treatment given, CT imaging can localize the presence of an abscess in the tonsillar space as opposed to an abscess encased in the tonsillar capsule, which is indicated by an ITA and is characterized by a central area of hypoattenuation and surrounding rim enhancement [9]. Discriminating between tonsillitis and ITA solely via oral examination poses challenges as both conditions manifest tonsillar swelling. Thus, ITAs can be distinctly discerned from tonsillitis even acute purulent tonsillitis through CT imaging [9]. Tonsillitis cases typically exhibit prominently swollen tonsils on CT without evidence of abscess formation [9]. In this case, the presentation resembles the peritonsillar abscess, and incision and drainage at the right peritonsillar region were unnecessary at the initial presentation. Misdiagnosis of ITA caused inappropriate treatment and persistent symptoms. Therefore, the CECT neck was done because of unresolved symptoms, which helped achieve a definite diagnosis: right unilateral ITA.

The surgical management of ITAs, including tonsillectomy and needle aspiration, has only been recorded in a small number of studies [8,10,11]. In this case, the primary strategy was needle aspiration of the abscess using an 18G needle in conjunction with intravenous antibiotic therapy. This primary modality was less invasive, cheap, easy, and less painful. The remaining pus drained more easily from the lumen at the aspirated site in the following days without incision and drainage. Compared to a surgical incision, this method has demonstrated less discomfort and bleeding after the procedure. However, post-procedural bleeding is a possible consequence of needle aspiration. This case had minimal post-procedural bleeding that stopped on its own without the need for medical attention. When placing the needle, care should be given to minimize the chance of inadvertently puncturing the major blood vessel if the needle is inserted too deeply [12]. Persistent infection can occur following this procedure. Therefore, the pus must be sent for culture and sensitivity analysis and treated with antibiotics according to their sensitivity. It is important to administer antibiotics before and after the procedure to lower the likelihood of a persistent infection.

## Conclusions

In conclusion, although ITA is seldom encountered, it should be considered a differential diagnosis of tonsillitis, peritonsillitis, or peritonsillar abscess. The symptoms and signs of tonsillitis, peritonsillitis, or peritonsillar abscesses are difficult to differentiate; therefore, imaging such as a CT scan can help to localize the collection of abscesses. A CT scan is not routinely done; however, during an unclear and uncertain diagnosis like in this case, it can be helpful to get the proper diagnosis. An intravenous antibiotic and needle aspiration of the ITA via the tonsillar parenchyma with an 18G needle in this case had emerged as an efficient main therapeutic strategy without surgical intervention.
